# Hearing Preservation After Segmental Semicircular Canal Destruction: A Report of Two Rare Cases

**DOI:** 10.1002/ccr3.72505

**Published:** 2026-04-10

**Authors:** Toru Miwa, Hideaki Ogita, Keishi Ueda, Akihito Tarui, Yasunori Asai, Taro Fujikawa, Nobuhiro Hakuba

**Affiliations:** ^1^ Department of Otolaryngology Teikyo University Hospital, Mizonokuchi Kawasaki Japan

**Keywords:** cartilage reconstruction, cholesteatoma, hearing preservation, labyrinthine fistula, semicircular canal fistula

## Abstract

Semicircular canal fistulas are a complication of cholesteatomatous otitis media. In extremely rare cases, extensive destruction of the bony labyrinth may result in loss of approximately one‐third of the semicircular canal arc, often with wide exposure and partial injury to the membranous labyrinth, thereby increasing the risk of profound sensorineural hearing loss. Complete eradication of the cholesteatoma while preserving inner ear function remains challenging. Here, we report two rare cases of segmental loss of the semicircular canal arc in which useful hearing was retained. Case 1 involved a 64‐year‐old man, in whom cholesteatoma erosion destroyed a continuous segment of the lateral semicircular canal, effectively eliminating approximately one‐third of the ring structure. After complete cholesteatoma removal, the entire defective segment was sealed with perichondrium and thin cartilage, under continuous saline irrigation and supplemented with postoperative steroid therapy. The fistula sign resolved, the bone conduction thresholds remained stable, and conductive hearing improved. Case 2 involved a 45‐year‐old man who underwent tympanoplasty. The intraoperative anatomical misorientation resulted in the unintended removal of a substantial portion of the posterior semicircular canal ring. Multilayer closure using the cartilage and fascia was performed while carefully preventing dehydration of the exposed membranous labyrinth. Postoperative audiometry showed retention of useful hearing at follow‐up in both cases, although Case 2 showed a mild early postoperative decline in bone conduction thresholds. These cases suggest that even in exceedingly rare situations involving segmental semicircular canal loss, prompt and meticulous surgical management may help preserve hearing. Important considerations include underwater dissection, avoidance of direct suction, multilayer cartilage‐based reconstruction, and perioperative steroid therapy.

## Key Clinical Message

Prompt recognition, gentle handling of the exposed labyrinth, and meticulous multilayer reconstruction may help preserve useful hearing in selected cases of severe semicircular canal injury.

## 1 Introduction

A labyrinthine fistula is a condition in which the bony wall of the labyrinth is destroyed by cholesteatomatous otitis media, chronic otitis media, trauma, or iatrogenic injury, resulting in abnormal communication between the inner ear and surrounding structures. Semicircular canal fistulas are the most common type, accounting for approximately 75% of all labyrinthine fistulas. The lateral semicircular canal is most frequently affected by chronic bone resorption and inflammatory changes associated with cholesteatoma. Clinically, fistulas may cause vertigo, a positive fistula sign, and progressive sensorineural hearing loss. Therefore, early diagnosis and careful surgical management are essential.

Surgical management of cholesteatoma‐related semicircular canal fistulas involves two competing goals: complete eradication of the cholesteatoma and preservation of auditory and vestibular functions. During cholesteatoma excision involving a fistula, techniques that minimize direct suction, allow dissection under saline irrigation, and preserve the membranous labyrinth are generally considered important to reduce additional inner ear injury [1].

Similar principles have been recommended for semicircular canal injuries caused by anatomical disorientation during middle ear surgery. The same membrane‐preserving technique under saline irrigation is recommended to reduce this risk [2]. Various materials, including fascia, bone pate, and cartilage, have been used for fistula closure, and postoperative hearing outcomes appear to depend largely on fistula size and the extent of membranous labyrinth preservation [3].

Labyrinthine fistulas are also classified according to the extent of the bony and membranous labyrinth involvement. According to the Milewski and Dornhoffer classifications, the most severe lesions involve the membranous labyrinth. Tomasoni et al. [4], identified semicircular canal fistulas in 947 of 13,554 middle ear surgeries (approximately 7%), with 90% involving the lateral semicircular canal and only 11% presenting as multiple fistulas. Cases in which the membranous labyrinth was preserved demonstrated significantly higher rates of hearing preservation, with an odds ratio of 4.56 [4]. However, cases involving segmental loss of the semicircular canal ring with two discrete fistulous openings are extremely rare, and detailed reports remain scarce. As such, lesions often indicate extensive disease and carry a high risk of membranous labyrinth injury; preserving auditory and vestibular functions is particularly challenging [3].

Here, we report two rare cases of extensive semicircular canal destruction requiring surgical repair, in which useful hearing was preserved. We describe the intraoperative findings, reconstruction strategies, and postoperative outcomes and discuss the surgical factors that may have contributed to hearing preservation. Written informed consent for the publication of case details and images was obtained from all the patients.

## 2 Case Presentation

### 2.1 Case 1

#### 2.1.1 Case History/Examination

**FIGURE 1:**
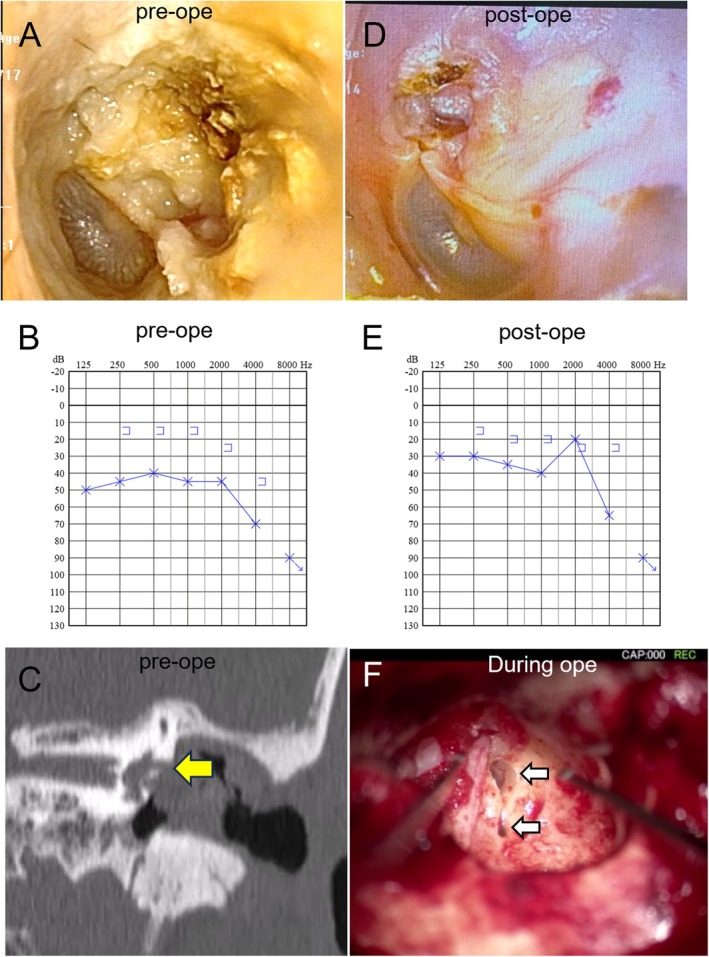
**Case 1**. (A) Preoperative tympanic membrane findings. (B) Preoperative audiometry. (C) Preoperative computed tomography findings. The yellow arrow indicates the bony defect in the lateral semicircular canal. (D) Postoperative tympanic membrane findings. (E) Postoperative audiometry findings. (F) Intraoperative findings. The white arrow indicates the bony defect in the lateral semicircular canal.

A 64‐year‐old man presented with transient rotational vertigo triggered by pressure applied to the left ear. He had no history of otological surgery. Otorrhea was absent, tinnitus was mild, and no signs of fever or meningeal irritation were observed. Otoscopic examination revealed retraction of the tympanic membrane with a cholesteatoma located in the posterosuperior quadrant (Figure 1A). The fistula sign was positive, and left‐beating horizontal nystagmus was elicited by negative pressure stimulation of the external auditory canal. Pure‐tone audiometry demonstrated an elevated air conduction threshold of 50.0 dB (four‐frequency average, 0.5, 1, 2, and 4 kHz), whereas bone conduction thresholds were preserved (Figure 1B). Temporal bone computed tomography (CT) revealed extensive bony destruction from the cholesteatoma, resulting in a partial defect of the lateral semicircular canal with surrounding soft tissue density (Figure 1C).

#### 2.1.2 Differential Diagnosis

Based on pressure‐induced vertigo, a positive fistula sign, and CT evidence of bony erosion, a labyrinthine fistula secondary to cholesteatoma was considered the primary diagnosis. Alternative diagnoses, including Ménière's disease and vestibular neuritis, were considered less likely, given the preserved bone conduction thresholds and radiologic findings.

#### 2.1.3 Conclusion and Results (Outcome and Follow‐Up)

Canal wall‐down mastoidectomy with canal wall reconstruction and type IIIc tympanoplasty were performed. Intraoperatively, approximately one‐third of the lateral semicircular canal ring was found to be continuously destroyed by the cholesteatoma. After removing the cholesteatoma matrix, the auricular cartilage was harvested, thinned, and used for ossicular reconstruction with a prosthesis (Apaceram P; HOYA Technosurgical Corporation, Tokyo, Japan), followed by a dome‐shaped tympanic membrane reconstruction. The matrix overlying the exposed semicircular canal was carefully removed using forceps, with a dexamethasone‐soaked cotton pledget placed beneath it, under continuous saline irrigation. This revealed a segmental bony defect involving approximately one‐third of the canal arc, with partial involvement of the membranous labyrinth, corresponding to the most severe end of the Milewski and Dornhoffer classification (Figure 1F). Direct suctioning and desiccation of the exposed labyrinth were avoided. Hydrodissection was not performed directly at the fistula site. The fistula was sealed using the harvested perichondrium, and two thin slices of cartilage were secured with fibrin glue. The fistula closure was completed within approximately 10 min. The surgical procedure is demonstrated in Video S1.

Postoperatively, severe rotational vertigo and irritative nystagmus persisted until postoperative day 3. A tapered 7‐day course of prednisolone (starting at 100 mg; 100–100–80‐80‐60‐40‐20) was administered postoperatively as an institutional treatment for a suspected inner ear insult. Assisted ambulation was initiated on postoperative day 4. Vertigo gradually improved, transitioning to mild unsteadiness by postoperative week 2. Independent ambulation was achieved after 1 month, and the fistula signs resolved. Otorrhea ceased (Figure 1D), and postoperative audiometry demonstrated preservation of bone conduction thresholds with improvement in the air conduction component at follow‐up (Figure 1E). Data summary was shown in Table 1.

**TABLE 1:** Clinical, operative, and audiological comparison of two cases of segmental semicircular canal destruction.

Variable	Case 1	Case 2
Age/sex	64‐year‐old man	45‐year‐old man
Underlying disease/setting	Cholesteatomatous otitis media	Chronic otitis media; intraoperative iatrogenic injury during tympanoplasty/mastoid surgery
Canal involved	Lateral semicircular canal	Posterior semicircular canal
Estimated defect size	Approximately one‐third of the canal arc	Approximately one‐third of the canal arc
Preoperative fistula sign	Positive	Negative
Vestibular findings	Pressure‐induced rotational vertigo; left‐beating horizontal nystagmus with negative pressure stimulation	No preoperative vestibular symptoms; mild postoperative disequilibrium at 1 week
Preoperative CT findings	Partial defect of the lateral semicircular canal with surrounding soft tissue density	No preoperative semicircular canal bony defect
Membranous labyrinth status	Wide exposure with partial injury/partial involvement; no complete transection	Extensive lesion involving the membranous labyrinth; partial disruption without complete transection
Classification interpretation	Most severe end of the Milewski and Dornhoffer classification	Most severe end of the Milewski and Dornhoffer classification
Surgical context	Cholesteatoma erosion identified intraoperatively	Intraoperative anatomical disorientation with inadvertent canal opening
Direct suction near fistula	Strictly avoided	Avoided in the immediate vicinity of the exposed labyrinth
Continuous saline irrigation	Yes	Yes
Hydrodissection	Not used directly at the fistula site	Not described
Closure/reconstruction method	Perichondrium + two thin cartilage slices + fibrin glue	Fascia + single thin cartilage plate + fibrin glue
Time required for fistula closure	Approximately 10 min	Approximately 5 min
Perioperative steroids	Yes; postoperative prednisolone taper for 7 days (starting at 100 mg/day)	No
Preoperative AC threshold/PTA	AC; 50.0 dB	AC; 32.5 dB
Preoperative BC threshold/PTA	BC; 25.0 dB	BC; 7.5 dB
Postoperative AC outcome	AC; 40.0 dB (6 months)	AC; 62.5 dB (2 weeks) AC; 18.75 dB (2 years)
Postoperative BC outcome	BC; 22.5 dB (6 months)	BC; 28.75 dB (2 weeks) BC; 6.25 dB (2 years)
Otorrhea outcome	Resolved	Resolved
Follow‐up duration	6 months	2 years
Final hearing outcome	Useful hearing retained	Useful hearing retained

### 2.2 Case 2

#### 2.2.1 Case History/Examination

**FIGURE 2:**
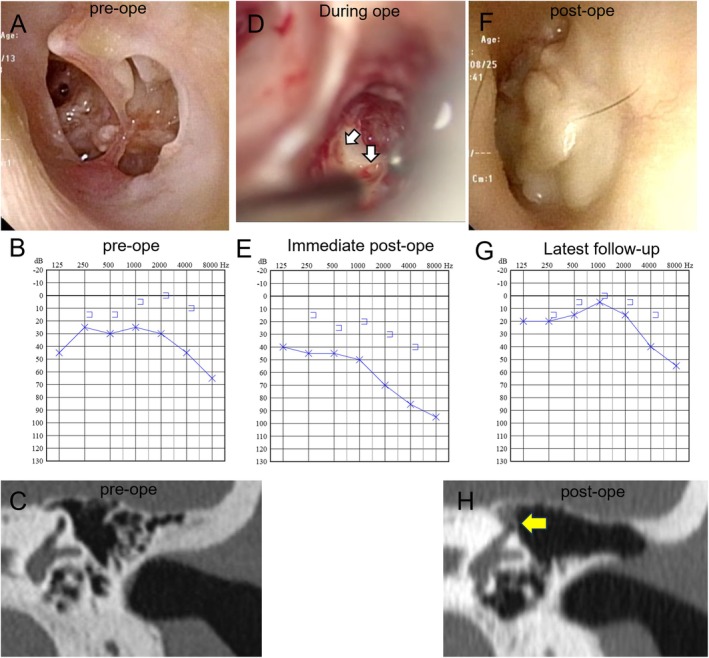
**Case 2**. (A) Preoperative tympanic membrane finding. (B) Preoperative audiometry. (C) Preoperative computed tomography findings. (D) Intraoperative findings. The white arrow indicates the bony defect in the posterior semicircular canal. (E) Postoperative audiometry after 2 weeks. (F) Postoperative tympanic membrane finding. (G) Postoperative audiometry after 2 years. (H) Postoperative computed tomography findings. The yellow arrow indicates the cartilage‐covered bony defect of the posterior semicircular canal.

A 45‐year‐old man with recurrent otitis media since childhood presented with recurrent otorrhea and aural fullness. Preoperative examination revealed no signs of a fistula, and temporal bone CT showed no evidence of semicircular canal bony defects. An otoscopic evaluation revealed a large perforation of the pars tensa (Figure 2A). Pure‐tone audiometry revealed elevated air conduction thresholds with preserved bone conduction hearing (Figure 2B). CT imaging revealed no significant soft tissue density within the tympanic cavity (Figure 2C).

#### 2.2.2 Differential Diagnosis

The primary preoperative diagnosis was chronic otitis media with tympanic membrane perforation. A labyrinthine fistula was not suspected preoperatively because of the absence of vestibular symptoms, negative fistula signs, and normal CT findings.

#### 2.2.3 Conclusion and Results (Outcome and Follow‐Up)

Intraoperative disorientation makes it difficult to identify the anatomical landmarks of the semicircular canals during surgery. The posterior semicircular canal was inadvertently opened while opening the mastoid air cells, resulting in excision of approximately one‐third of the canal arch. This degree of injury corresponded to an extensive lesion involving the membranous labyrinth and was interpreted as representing the most severe end of the Milewski and Dornhoffer classification (Figure 2D). Large volumes of irrigation fluid and continuous saline irrigation were used to prevent the desiccation of the membranous labyrinth. A saline‐soaked gauze was placed in the mastoid cavity, and the auricular cartilage and fascia were harvested. The cartilage was thinned, and the fascia was stretched and dried. Direct suction was avoided in the immediate vicinity of the exposed labyrinth. The fistula was closed using fascia, followed by coverage with a single thin cartilage plate secured using fibrin glue. Closure was completed within approximately 5 min. The surgical procedure is demonstrated in Video S2.

Postoperatively, the patient experienced mild disequilibrium at 1 week, which resolved rapidly with observation alone. Audiometry at 2 weeks showed a mild decline in bone conduction thresholds (Figure 2E). At the 2‐year follow‐up, the tympanic membrane perforation had closed with no otorrhea (Figure 2F), recurrence, or progressive hearing loss (Figure 2G). CT confirmed closure of the fistula with soft tissue density (Figure 2H), and useful hearing was retained. Data summary was shown in Table 1.

## 3 Discussion

We present two rare cases of semicircular canal destruction involving the segmental loss of approximately one‐third of the canal arc. In Case 1, extensive cholesteatomatous erosion resulted in wide exposure with partial injury of the membranous labyrinth of the lateral semicircular canal. In Case 2, intraoperative disorientation led to inadvertent fenestration and segmental excision of the posterior semicircular canal. Although the membranous labyrinth was partially disrupted in both cases, complete transection did not occur, and rapid multilayer reconstruction under continuous irrigation may have helped preserve useful hearing.

The primary goals of treating cholesteatoma‐associated semicircular canal fistulas are complete eradication of the cholesteatoma matrix and preservation of inner ear function, particularly hearing. These goals are often in conflict with each other. Excessive manipulation or direct suction at the fistula site can damage the membranous labyrinth or induce leakage of perilymph or endolymph, resulting in irreversible postoperative sensorineural hearing loss. Therefore, fundamental surgical principles are essential, including addressing the fistula at the end of the dissection, avoiding direct suction, and performing maneuvers under saline irrigation [2]. In these cases, strict adherence to these principles may have facilitated cholesteatoma removal while maintaining postoperative auditory function.

Disorientation during surgery owing to missing anatomical landmarks increases the risk of labyrinthine injury. In such cases, both the suction‐generated negative pressure and drill‐induced vibration can transmit force to the inner ear. Therefore, frequent irrigation and repeated anatomical confirmation are valuable strategies to prevent iatrogenic membranous labyrinth damage.

The severity of semicircular canal fistulas is commonly categorized using the Milewski and Dornhoffer classification (types I–III) [5]. Postoperative hearing preservation is highest in type IIa–IIb lesions, in which the membranous labyrinth remains intact [4]. Consistent with this finding, several predictors of favorable auditory outcomes were identified: (1) membranous labyrinth preservation (odds ratio, 4.56), (2) isolated lateral semicircular canal involvement (odds ratio, 3.52), (3) fistula diameter < 3 mm (odds ratio, 6.15), and (4) perioperative steroid administration (odds ratio, 7.87). Steroid therapy protects the bone conduction thresholds by suppressing postoperative inflammation and reducing labyrinthine edema.

In our cases, perioperative steroid administration was present in Case 1, whereas isolated lateral semicircular canal involvement was present only in Case 1. Thus, the concordance with these favorable predictors was incomplete. In addition, predictors 1 and 3, which are strongly associated with favorable hearing outcomes, were not fulfilled in either case. Despite these unfavorable prognostic features, postoperative auditory function was preserved, suggesting that rapid surgical manipulation and minimizing exposure time to the membranous labyrinth may help limit additional inner ear injury. Taken together, these cases support the importance of meticulous dissection under continuous irrigation, strict avoidance of suction, and prompt multilayer reconstruction using cartilage, fascia, or perichondrium.

Importantly, our cases demonstrate that even with extensive exposure and partial destruction of the membranous labyrinth, irreversible inner ear damage may sometimes be avoided if the duration of exposure is minimized and prompt reconstruction is performed. These observations emphasize the critical role of surgical precision and labyrinth‐protective strategies in preventing irreversible sensorineural hearing loss, particularly in complex fistulas involving segmental semicircular canal defects or iatrogenic fenestrations.

Various techniques and materials have been used for fistula closure. Some authors advocate rigid closure with bone dust or bone wax [6], whereas others support more flexible coverage with the fascia or perichondrium [7]. In our cases, multilayer reconstruction with cartilage, perichondrium, and fibrin glue yielded stable closure without postoperative recurrence or inner ear dysfunction. Favorable hearing outcomes following cartilage‐based reconstruction have been previously reported [8, 9, 10, 11]. Cartilage offers advantages such as excellent conformity to defects and elasticity, which may buffer pressure fluctuations within the labyrinth.

## 4 Conclusion

Semicircular canal fistulas with segmental bony destruction pose a significant challenge because of the high risk of membranous labyrinth injury and subsequent sensorineural hearing loss. In both cases presented here, successful outcomes relied on strict adherence to rapid, meticulous surgical management, including delaying fistula closure until the final stage, avoiding direct suction, maintaining continuous saline irrigation, and promptly performing multilayer reconstruction with cartilage or perichondrium. These findings indicate that even when partial destruction of the membranous labyrinth occurs, hearing preservation may be achieved through precise, swift surgical manipulation, combined with flexible cartilage‐based reconstruction and perioperative steroid therapy.

Taken together, our findings underscore the importance of precise anatomical orientation, gentle tissue handling, and continuous irrigation in minimizing inner ear injury and suggest that a minimally invasive, labyrinth‐preserving approach supplemented with perioperative steroids may help preserve hearing after challenging semicircular canal fistula surgery.

## Author Contributions

**Toru Miwa:** data curation, formal analysis, investigation, methodology, software, visualization, writing – original draft. **Hideaki Ogita:** methodology, resources. **Keishi Ueda:** resources, validation, writing – review and editing. **Akihito Tarui:** data curation, resources, validation, writing – review and editing. **Yasunori Asai:** conceptualization, resources, software, visualization. **Taro Fujikawa:** conceptualization, resources, supervision, validation, visualization, writing – review and editing. **Nobuhiro Hakuba:** conceptualization, investigation, methodology, project administration, supervision, validation, writing – review and editing.

## Funding

This study received no specific grants from any funding agency in the public, commercial, or not‐for‐profit sectors.

## Ethics Statement

The Ethics Committee of Teikyo University approved the study protocol (approval no. 19–074‐6), and the study was conducted in accordance with the Declaration of Helsinki.

## Consent

Written informed consent for the publication of case details and images was obtained from the patient.

## Conflicts of Interest

The authors declare no conflicts of interest.

## Supporting information


**Video S1** (Case 1). This placeholder represents the supplementary video demonstrating the surgical management of a semicircular canal fistula in Case 1. The video shows careful removal of the cholesteatoma matrix under continuous saline irrigation, avoidance of direct suction at the fistula site, and multilayer reconstruction using perichondrium and thin cartilage.

**Video S2** (Case 2). This placeholder represents the supplementary video demonstrating the intraoperative management of an iatrogenic semicircular canal injury in Case 2. The video shows preservation of the membranous labyrinth under continuous irrigation and multilayer reconstruction using fascia and cartilage.

## Data Availability

The data that support the findings of this study are available from the corresponding author upon reasonable request.
