# Supplementary figures and images for: Hearing Preservation After Segmental Semicircular Canal Destruction: A Report of Two Rare Cases
Source: Clin Case Rep. 2026 Apr 10;14(4):e72505. doi: 10.1002/ccr3.72505 (PMC13069008; doi:10.1002/ccr3.72505)

## Slide 1
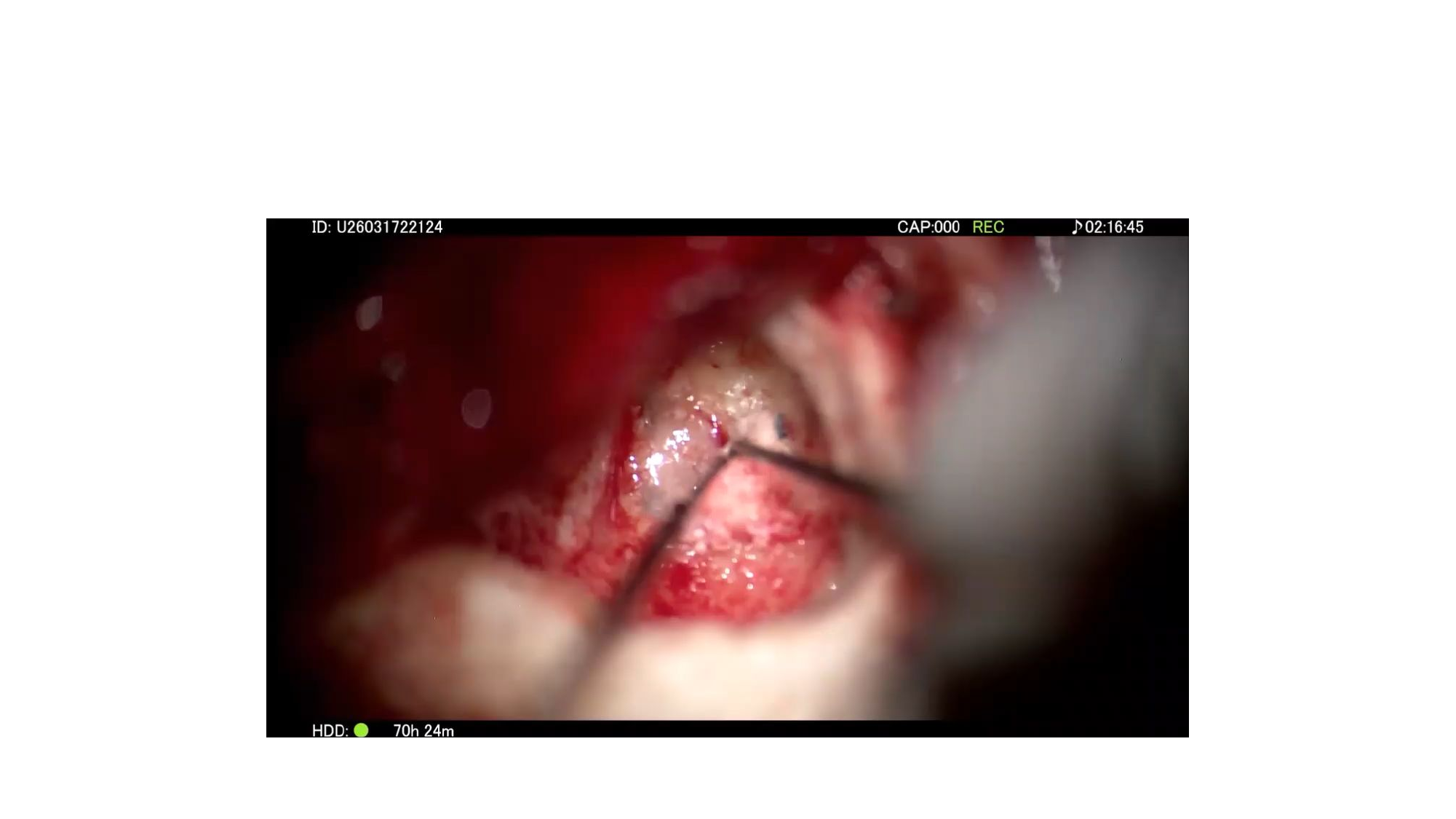

Supplement: Supplementary file 1 — Video S1 (Case 1). This placeholder represents the supplementary video demonstrating the surgical management of a semicircular canal fistula in Case 1. The video shows careful removal of the cholesteatoma matrix under continuous saline irrigation, avoidance of direct suction at the fistula site, and multilayer reconstruction using perichondrium and thin cartilage. [file CCR3-14-e72505-s001.pptx]

## Slide 1
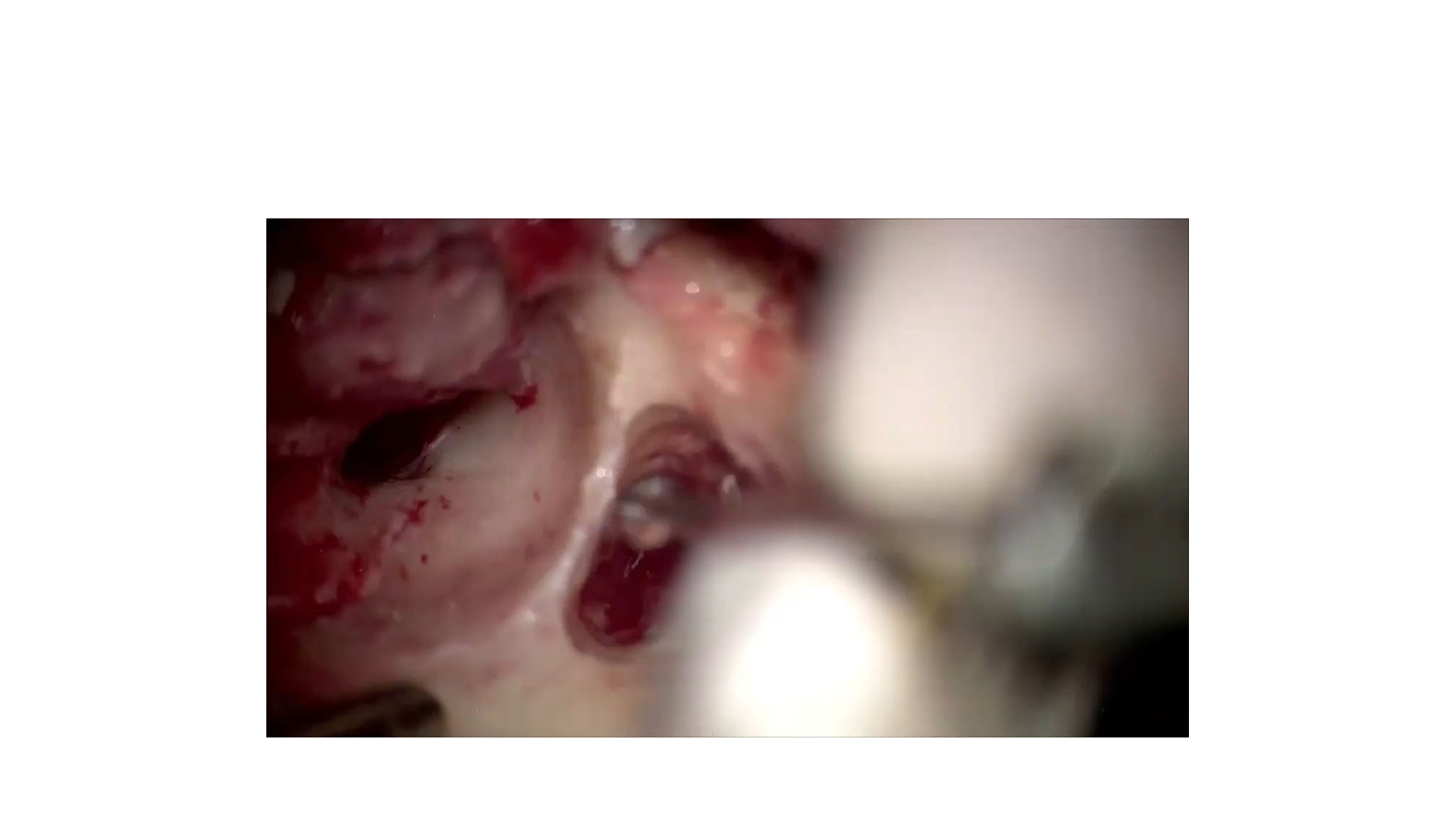

Supplement: Supplementary file 2 — Video S2 (Case 2). This placeholder represents the supplementary video demonstrating the intraoperative management of an iatrogenic semicircular canal injury in Case 2. The video shows preservation of the membranous labyrinth under continuous irrigation and multilayer reconstruction using fascia and cartilage. [file CCR3-14-e72505-s002.pptx]
